# Impedimetric Biosensors for Detecting Vascular Endothelial Growth Factor (VEGF) Based on Poly(3,4-ethylene dioxythiophene) (PEDOT)/Gold Nanoparticle (Au NP) Composites

**DOI:** 10.3389/fchem.2019.00234

**Published:** 2019-04-16

**Authors:** Minsoo Kim, Raymond Iezzi, Bong Sup Shim, David C. Martin

**Affiliations:** ^1^Department of Materials Science and Engineering, University of Delaware, Newark, DE, United States; ^2^Department of Ophthalmology, Mayo Clinic, Rochester, MN, United States; ^3^Department of Chemical Engineering, Inha University, Incheon, South Korea

**Keywords:** VEGF (Vascular Endotelial Growth Factor), PEDOT (poly(3, 4-ethylenedioxythiophene)), biosensor, electrochemical deposition, impedance spectroscopy

## Abstract

In advanced forms of diabetic retinopathy, retinal vascular occlusive disease and exudative age-related macular degeneration, vision loss is associated with elevated levels or extravasation of vascular endothelial-derived growth factor (VEGF) into the retina, vitreous, and anterior chamber of the eye. We hypothesize that point-of-care biosensors, capable of rapidly and precisely measuring VEGF levels within the eye will assist clinicians in assessing disease severity, and in establishing individualized dosing intervals for intraocular anti-VEGF injection therapy. An impedance biosensor based on a poly(3,4-ethylenedioxythiophene) (PEDOT)/gold nanoparticle (Au NP) composite was developed for detecting VEGF. PEDOT with Au NP was electrochemically deposited on three different medical electrode sensor designs: free-standing pads, screen printed dots, and interdigitated micro-strip electrodes. Anti-VEGF antibody was covalently immobilized on the surface of the polymer films through attachment to citrate-functionalized Au NPs, and the resulting composites were used to detect VEGF-165 by electrochemical impedance spectroscopy (EIS). The PEDOT-Au NP composite materials were characterized using optical microscopy, SEM/EDS, FIB, TEM, and STEM techniques. Among the different micro-electrodes, the interdigitated strip shape showed the best overall film stability and reproducibility. A linear relationship was established between the charge transfer resistance (*R*_*ct*_) and VEGF concentration. The detection limit of VEGF was found to be 0.5 pg/mL, with a correlation coefficient of 0.99 ± 0.064%. These results indicate that the proposed PEDOT/Au NP composites can be used in designing low-cost and accurate VEGF biosensors for applications such as clinical diagnosis of VEGF-mediated eye disease.

## Introduction

VEGF-mediated eye disease is associated with several causes of severe vision loss due to exudative Age-related Macular Degeneration (AMD), advanced diabetic retinopathy, and retinal vascular occlusive disease. These three disease groups may often be successfully treated or controlled with long-term intravitreous injection therapy using anti-VEGF drugs such as bevacizumab, an engineered antibody to VEGF-165, ranibizumab, and engineered antibody fragment with enhanced binding affinity to VEGF-165, and aflibercept, an anti-VEGF fusion protein (Hernandez et al., 2018). These drugs are aimed at reducing intraocular levels of biologically-active VEGF, a signaling protein important in vasculogenesis and angiogenesis that promotes the growth and survival of vascular endothelial cells. VEGF is known to exist in five different isoforms containing 121, 145, 165, 189, and 206 amino acids, respectively (Ferrara, 2004). Both VEGF-121 and VEGF-165 are found in neovascular membranes in patients with AMD (Rakic et al., 2003). VEGF-165 is the predominant isoform associated with increased vascular permeability and retinal vascular proliferation (Adamis and Shima, 2005). The aqueous concentration of VEGF in the eyes of the AMD patients ranged from 74.5 to 521.6 pg/mL (Cabral et al., 2017). However, anti-VEGF treatments using anti-angiogenic drugs require repetitive and costly intravitreal injections for patients (Maguire, 2012). Thus, the precise amount of anti-VEGF drug that should be injected and the total number of treatments required could be better controlled by detecting the VEGF concentration in patient's retina. This potential ability to accurately measure VEGF concentrations should substantially reduce the financial burden for patients as well the potential over/under-dose side effects by the anti-VEGF drug treatments. While an implantable biosensor would be highly desirable for continuous *in situ* monitoring during the treatment, components of biosensor technologies still need to be developed including a non-cytotoxic and conductive coating material with a large surface area, an ability to be coated onto a needle-shaped electrode, and the capability of stable, non-destructive measuring.

Biosensors selectively detect the presence or concentrations of a specific biological target by mechanisms such as optical, electrochemical, thermometric, piezoelectric, or magnetic transductions (Potyrailo and Mirsky, 2009). Electrochemical biosensors measure a biochemical interaction between a bioactive substance on a sensor and a biomarker, making it possible to convert the concentrations of VEGF to a quantifiable electrochemical signal. The change of electrochemical signals is coupled to immobilized VEGF on the surface of the sensor. Impedimetric biosensors measure the changes in charge conductance and capacitance at the sensor surface as the selective binding of the target occurs. Here, we used Electrochemical Impedance Spectroscopy (EIS) to measure the impedance changes associated with varying VEGF concentrations in the analyte. EIS has been previously used for monitoring the healthy conditions of animal tissues *in vivo* (Dean et al., 2008) as well as for detecting a wide variety of biomolecules such as proteins (Smiechowski et al., 2006), DNA (Park and Park, 2009), small molecules (Kara et al., 2010), and direct cell-based assays (Mishra et al., 2005). While EIS sensors for VEGF detection have been introduced by utilizing a series of anti-VEGF aptamers (Qureshi et al., 2015; Shamsipur et al., 2015), there are still serious limitations of previous designs for intraocular AMD treatment. Remaining issues include the ability to measure VEGF over a wide range of concentrations, the ability to have an implantable electrode design, and chemical and biological stability.

Conjugated polymers are a unique set of polymeric materials that have intriguing combinations of properties such as electronic and ionic conductivities, and biocompatibility. Conjugated conducting polymers can be polymerized either chemically or electrochemically (Inzelt, 2012). One of the most well-studied of these materials is poly(3,4-ethylenedioxythiophene) (PEDOT)/polystyrene sulfonate (PSS), which is currently used in a variety of organic electronic and bioelectronic applications. PEDOT can be doped and entangled by anionic molecules or polymers because conjugated polymers including PEDOT are p-type semiconductors. While neighboring anionic dopants could not ionize PEDOT, they draw electronic clouds so that PEDOT hold increased concentration of delocalized mobile hole conductors along the conjugated structures. These dopants can be replaced by any form of anions during the polymerization process of EDOT. In this case, citric acid has been used to dope PEDOT as well as to stabilize Au NPs on the composite of PEDOT with improved biofunctionality compared to PSS. Commercially available PEDOT/PSS is polymerized chemically with an oxidizing agent (Elschner et al., 2010). Another method is electrochemical polymerization. An applied electrical current causes irreversible oxidation of the monomer through series of reactions, and resulting in deposition of the polymers on an electrode (El-Abdallah, 2014). A nano- or micro-scale thin polymer film can be deposited on an electrode surface by varying amount of monomers, processing time, and applied electrical charge (Olowu et al., 2010). This conformable deposition is also applicable to coat non-flat electrodes. In designing materials for an EIS biosensor, conjugated polymers can improve the sensitivity and selectivity by which bioactive recognition elements are immobilized on the electrode. The conducting polymers may act as a molecular cable for the direct electron transfer processes between the recognition elements and the electrode surface (Bard et al., 2012). Combined with all these unique properties, a few selected conducting polymers are also biocompatible. Recently, Miriani et al. (2008) reported that PEDOT is not cytotoxic to cells and successfully supports cellular proliferation and differentiation. In addition to its non-cytotoxicity, the rough surface morphology, and charge conduction properties of PEDOT would also be beneficial as a bio-electrode coating *in vivo* system. We have designed a PEDOT-based impedimetric VEGF biosensor via electropolymerization. To improve its binding to the antibody by overcoming the inherent lack of surface functionality of PEDOT, we synthesized a PEDOT/citrate-capped Au nanoparticle (NP) composite. We hypothesized that the PEDOT would interact strongly with Au NPs (Häkkinen, 2012), entrapping them into the PEDOT polymer film during the elctropolymerization (Pan et al., 2015). The citrate-capped Au NPs would enable the resulting film to bind to the antibody of interest with covalent surface reactions. As a proof of principle, we have investigated three types of medical grade electrode designs including three-dimensional freestanding metal pads, screen-printed dots, and inter-digitated micro-strip electrodes. We confirmed the conformable electrochemical deposition of polymer/Au NP composites onto these various electrodes and their functionalization with the VEGF antibody. We used EIS techniques to examine the response of the sensors to solutions with variations of VEGF concentration, and fit the data to three-element equivalent circuit models. We found that the charge-transfer resistance (*R*_*ct*_) could be directly correlated with VEGF concentration in the analyte. Finally, we conclude with the implications and future directions for this work.

## Experimental

### Materials

3,4-ethylenedioxylthiophene (EDOT, >97%) monomer was obtained from Sigma–Aldrich (St. Louis, MO, USA). Citrate-capped Au NPs with an average diameter of 10 nm and concentration of 40–50 μg/mL were obtained from NN-Labs (Fayetteville, AR, USA). Ranibizumab (Trade name: Lucentis, MW: 48 kDa), a monoclonal VEGF antibody without a fragment crystallized (Fc) region was obtained from the Mayo Clinic (Rochester, MN., USA). Human recombinant VEGF-165 was purchased from Sigma–Aldrich (St. Louis, MO, USA). The contents of anti-VEGF antibody and VEGF-165 were reconstituted using ultrapure water. Anti-VEGF antibody and VEGF-165 were stored at 4°C and at −20°C, respectively, prior to use. Potassium hexacyanoferrate (III) (K_3_Fe(CN)_6_, >99%), potassium hexacyanoferrate (II) trihydrate (K_4_Fe(CN)_6_, 98.5–102.0%), and potassium chloride (KCl, >99%) were purchased from Sigma–Aldrich (St. Louis, MO, USA). A redox probe solution for electrochemical measurements was prepared from an aqueous solution of 0.005 M [Fe(CN)_6_]^−3/−4^ in 0.1 M KCl at pH 7. 2-(N-morpholino)ethanesulfonic acid (MES) buffered saline packs were purchased from Thermo Fisher (San Jose, CA, USA). Cross-linkers including 1-ethyl-3-(3-dimethylaminopropyl)carbodiimide (EDC) and N-hydroxysuccinimide (NHS) were purchased from Pierce, Germany. Water was purified to a resistance of 18 MΩ/cm (Academic Milli-Q Water System; EMD Millipore, Billerica, MA, USA) and filtered through a 0.22 μm membrane filter prior to use.

As working electrodes, four types of electrodes were used; stainless steel free-standing pad electrodes for morphological characterization and biosensing, gold transmission electron microscopy (TEM) grids for TEM characterization, gold screen printed dot electrodes (Au SPEs), and gold interdigitated micro-stripe electrodes (Au IDEs). Stainless steel pad electrodes were purchased from Plastics One (Roanoke, VA, USA) and gold TEM grids were purchased from Electron Microscopy Sciences (Hatfield, PA, USA). Au SPEs with a working diameter of 1.6 mm (gold as a working electrode and a counter electrode, and Ag/AgCl as a reference electrode) and Au IDEs (two gold working and counter channels, each channel: 125 bands, dimension of bands/gaps: 10 μm) were purchased from Metrohm AG (Riverview, FL, USA). Platinum foil (99.999%) was used as an external counter electrode and a reference electrode was purchased from Alfa Aesar (Word Hill, MA, USA). An Ag/AgCl reference electrode (4 M KCl) was obtained from Fisher Scientific Accumet (Pittsburgh, PA, USA).

### Electrochemical Polymerization

All electrochemical experiments were carried out with an Autolab PGSTAT12 (Metrohm Autolab, Utrecht, The Netherlands) potentiostat/galvanostat with a frequency response analyzer (FRA) module. 0.005 M of EDOT was mixed with an equal volume of a commercial aqueous solution of Au NPs and the mixture was stirred for 5 min. The ratio of EDOT to Au solution was adjusted to 1:2 by volume. The polymerization was performed in a two-electrode cell with a diameter of 3 cm, where one of three types of electrodes was used as a working electrode, and an external platinum foil or a gold counter channel on an IDE was used as a counter and reference electrode. After the electrochemical polymerization was completed, the residual EDOT monomer and Au NPs were removed by gentle washing with deionized water. To prepare samples for scanning electron microscopy (SEM), electrochemical polymerization of PEDOT/Au NP composite on the stainless-steel electrode was carried out under galvanostatic conditions in a mixture of EDOT and Au NPs at a current density of 1 mA/cm^2^ for 30 min. To prepare samples for TEM, electrochemical polymerization of PEDOT/Au NP composite on the gold TEM grid was carried out under galvanostatic conditions in a mixture of EDOT and Au NPs at a current density of 0.1 mA/cm^2^ for 20 s. To perform biosensing, electrochemical polymerization of PEDOT/Au NP composite on the stainless-steel pad electrode, Au SPEs, and Au IDEs were carried out under galvanostatic conditions in a mixture of EDOT and Au NPs at a current density of 0.1–1 mA/cm^2^ for 1–30 min, 0.1–5 mA/cm^2^ for 1–10 min, and 0.1–0.2 mA/cm^2^ for 3–5 min, respectively.

### Morphological Characterization

Optical microscopy was conducted on a Nikon SMZ800 (Mager Scientific, Dexter, Michigan, USA). Digital images were acquired with a OMAX A3590U digital camera. For SEM and focused ion beam (FIB*)* measurements, an Auriga 60 crossbeam FIB-SEM instrument (Carl Zeiss, Oberkochen, Germany) was used. Augria 60 crossbeam FIB-SEM using an electron beam at the acceleration voltage of 3 kV and a gallium ion beam with a current of 4 nA and the acceleration voltage of 30 kV for ion beam milling to enable cross-sectional analysis was performed. FIB images were recorded at an incident angle of 52°. X-ray energy dispersive spectroscopy (XEDS) was performed on the SEM using Oxford Synergy X-MAX80 & EBSD. For TEM/Scanning Transmission Electron Microscopy (STEM), the samples were studied by a JEOL JEM-3010 transmission electron microscope with a 300 kV field emission gun.

### Anti-VEGF Immobilization and VEGF Binding

Two different strategies for the immobilization of antibodies were used for stainless steel electrodes and IDEs: electrostatic interaction for stainless steel electrodes and covalent binding for IDEs. 0.1 M MES solution with 0.9% sodium chloride at pH 4.7 was prepared by dissolving the saline powder to deionized water for physical immobilization of antibodies. It was performed by dipping the stainless-steel electrode in tubes containing 200 μL of 0.1 M MES solution with the anti-VEGF antibody and then the electrodes were incubated at room temperature for 2 h. EDC/NHS coupling agents were used for covalent immobilization of antibody on the electrode surface of IDEs. The terminal—COOH group of citrated stabilized Au NPs was activated using EDC/NHS. The PEDOT coated electrode was immersed in a solution of 0.4 M EDC and 0.1 M NHS for 1 h at room temperature. The resulting electrode was allowed to wash with deionized water and purge under a stream of argon gently. Fifty microliters of anti-VEGF antibody was dropped on the surface of Au IDEs with a pipette and the electrodes were incubated at room temperature for 2 h. All electrodes were then gently rinsed with deionized water to remove unreacted anti-VEGF antibodies on the electrode surface. Finally, 50 μL of VEGF at 0–500 pg/mL was dropped on the surface of electrodes and was incubated at room temperature in a closed container to prevent evaporation for 1 h. All electrodes were gently washed with deionized water again to remove unreacted VEGF.

### Electrochemical Measurements

Cyclic Voltammetry (CV) was carried out in 0.1 M KCl solution containing 5 mM ferry/ferrocyanide (1:1) as a redox component to characterize the deposition of polymer/metal composites on an IDE. Voltage was applied from −400 to 800 mV in a step size of 2 mV at a scan rate of 120 mV/s. EIS measurements for VEGF sensing were performed with an alternating sinusoidal signal of 10 mV amplitude and direct current (DC) potential was set to 0 mV. Impedance spectra were collected by scanning the frequency range from 1 to 10 kHz. The same redox couple was also used for impedance measurements. All impedance measurements were carried out in a Faraday cage to minimize external interference on the system. Both CV and EIS measurements were performed in a three-electrode configuration. The stainless-steel electrodes or IDEs coated PEDOT/Au NP composite were used as a working electrode while an external platinum foil was used as a counter electrode and an Ag/AgCl (in 4 M KCl) electrode was used as a reference electrode. The experimental spectra of EIS were fitted to an equivalent circuit using the Windows software ZView v3.4e (Scribner Associates).

## Results and Discussion

### Structural Characterization of Electrochemical PEDOT/Au NP Composite

**Figure 1:**
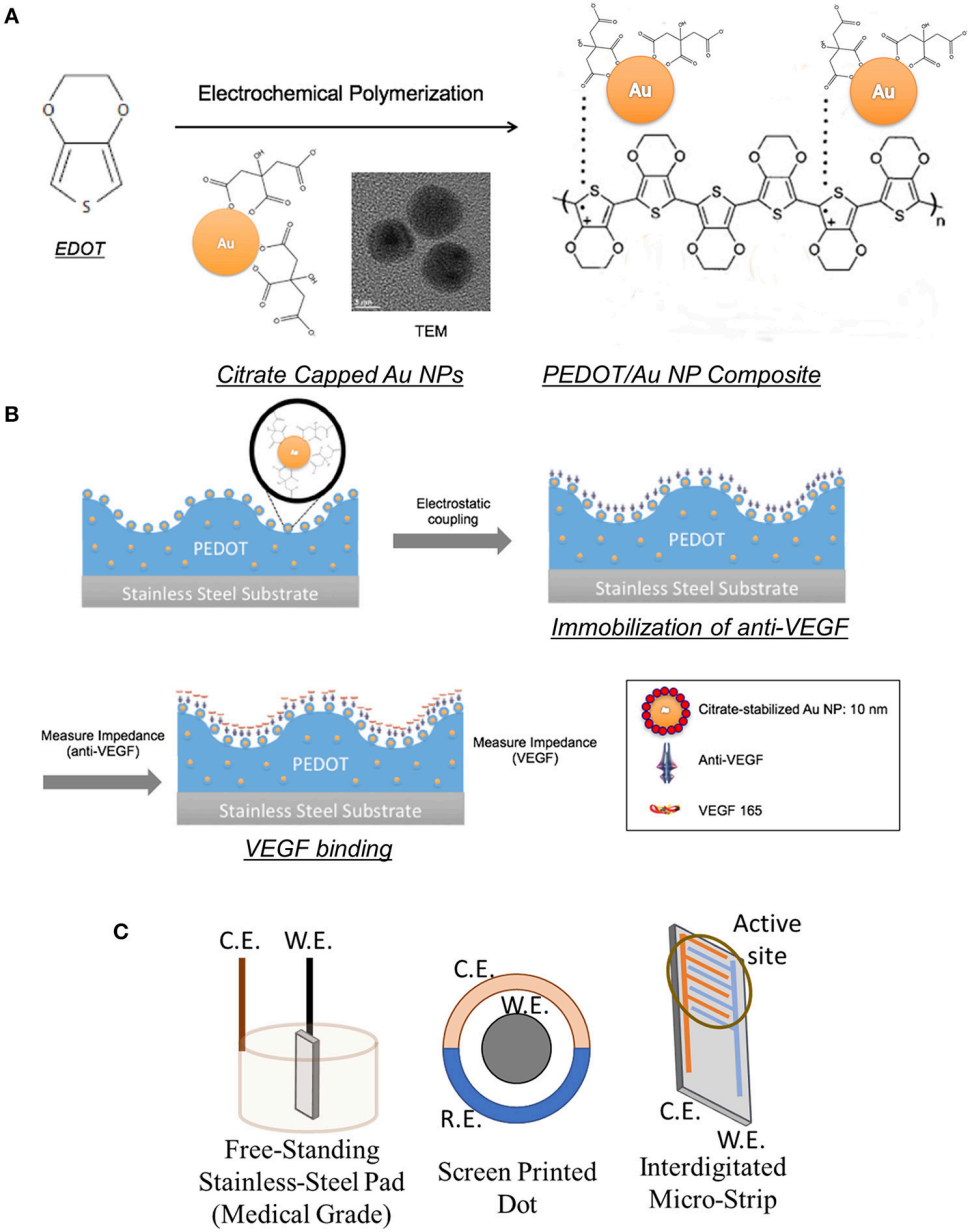
Schematics of PEDOT/Au NP composites for VEGF sensors: **(A)** Electrochemical polymerization of PEDOT/Au NP composites **(B)** Anti-VEGF immobilization and VEGF binding **(C)** Configurations of working (W.E.), counter (C.E.), and reference (R.E.) electrodes.

Schematics of electrochemical preparation of PEDOT/Au NP composites and selective detection VEGF 165 by anti-body binding are presented in Figure 1. The mediating Au NPs were also characterized by UV-visible spectroscopy and TEM as shown in Figure S1. The surface plasmon absorption band of Au NPs was observed at 525 nm. The average diameter of Au NPs was determined to be 10.6 ± 0.7 nm using TEM. The synthesis of PEDOT/Au NP composites was carried out on three different micro-electrodes by electrochemical polymerization; three-dimensional freestanding pad, screen-printed dot, and inter-digitated strip electrodes. The as-prepared sensor with 200 nm thickness of PEDOT/Au NP composite had poor sensitivity to detect tens to hundreds pg/mL of VEGF concentration accurately, which is required for treating average AMD patients clinically. To improve the sensitivity, aggregated Au NPs, on which antibodies were bound to citrate-capped surface via covalent bonding, were deposited on PEDOT binders. For achieving this complex material structure in a one pot synthesis, an excess amount of Au NP solution was mixed with EDOT solution prior to the electrochemical polymerization (1:1 to 1:3 volume ratio). The EDOT and Au NPs were polymerized together on the electrode as a composite and then excess Au NPs were still aggregated. This aggregated structure provides additional sensitivity, presumably due to electrical charge transfer through antigen/ antibody complex formation. We investigated a wide range of volume ratios of Au NP solutions to EDOT solutions during the electrochemical polymerization but the biosensors without aggregated gold layers showed a poor sensitivity to detect VEGF.

**Figure 2:**
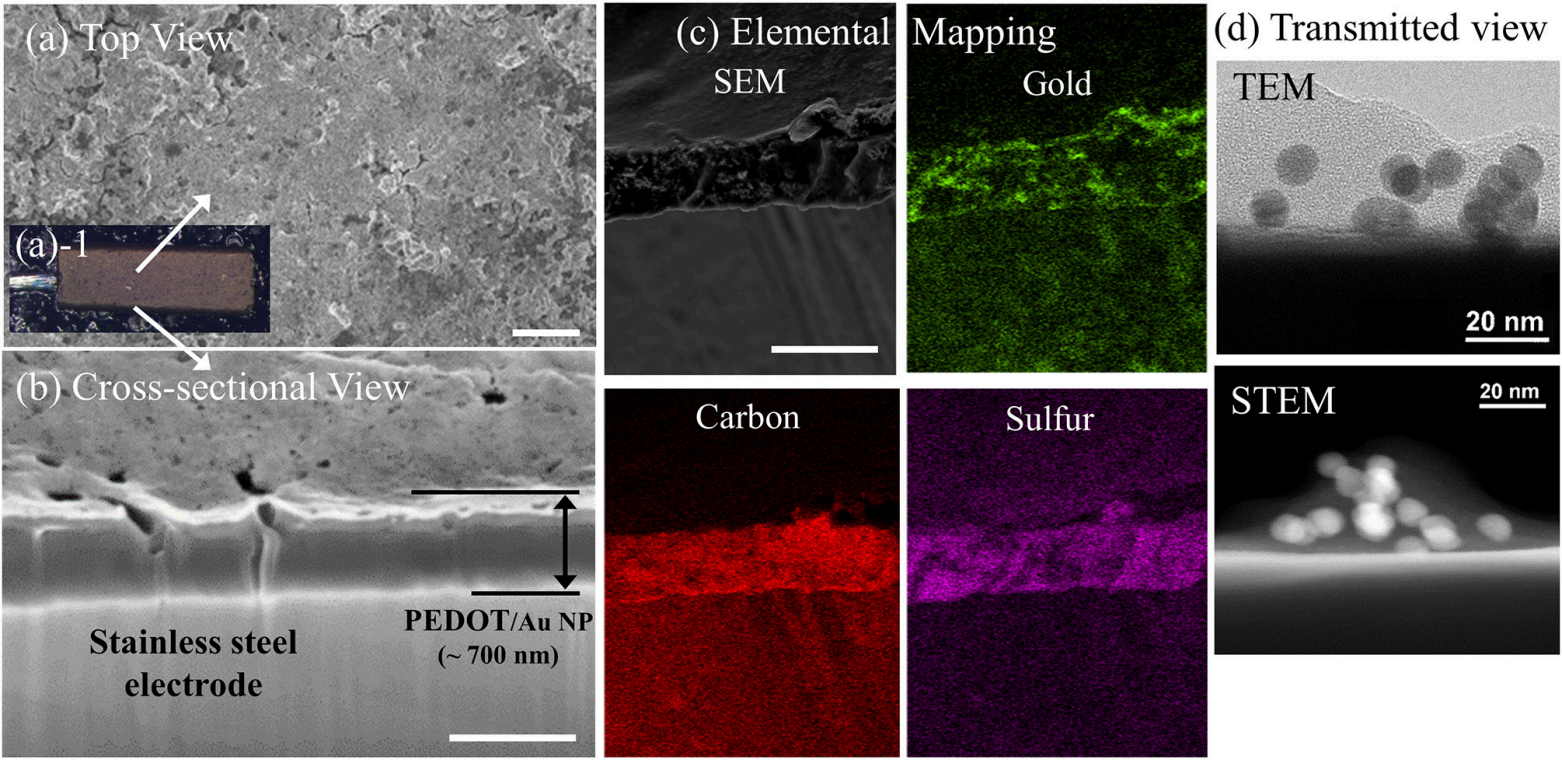
Images of PEDOT/Au NP composites on a free-standing pad electrode. **(a)** Top view, (a-1) **(a)** SEM (scale bar 1 μm) **(b)** optical microscopy **(c)** cross-sectional SEM images of PEDOT/Au NP composite and aggregated Au NPs on a pad electrode (scale bar 500 nm) **(d)** Transmitted images of a PEDOT/Au NP composite by TEM and STEM.

**Figure 3:**
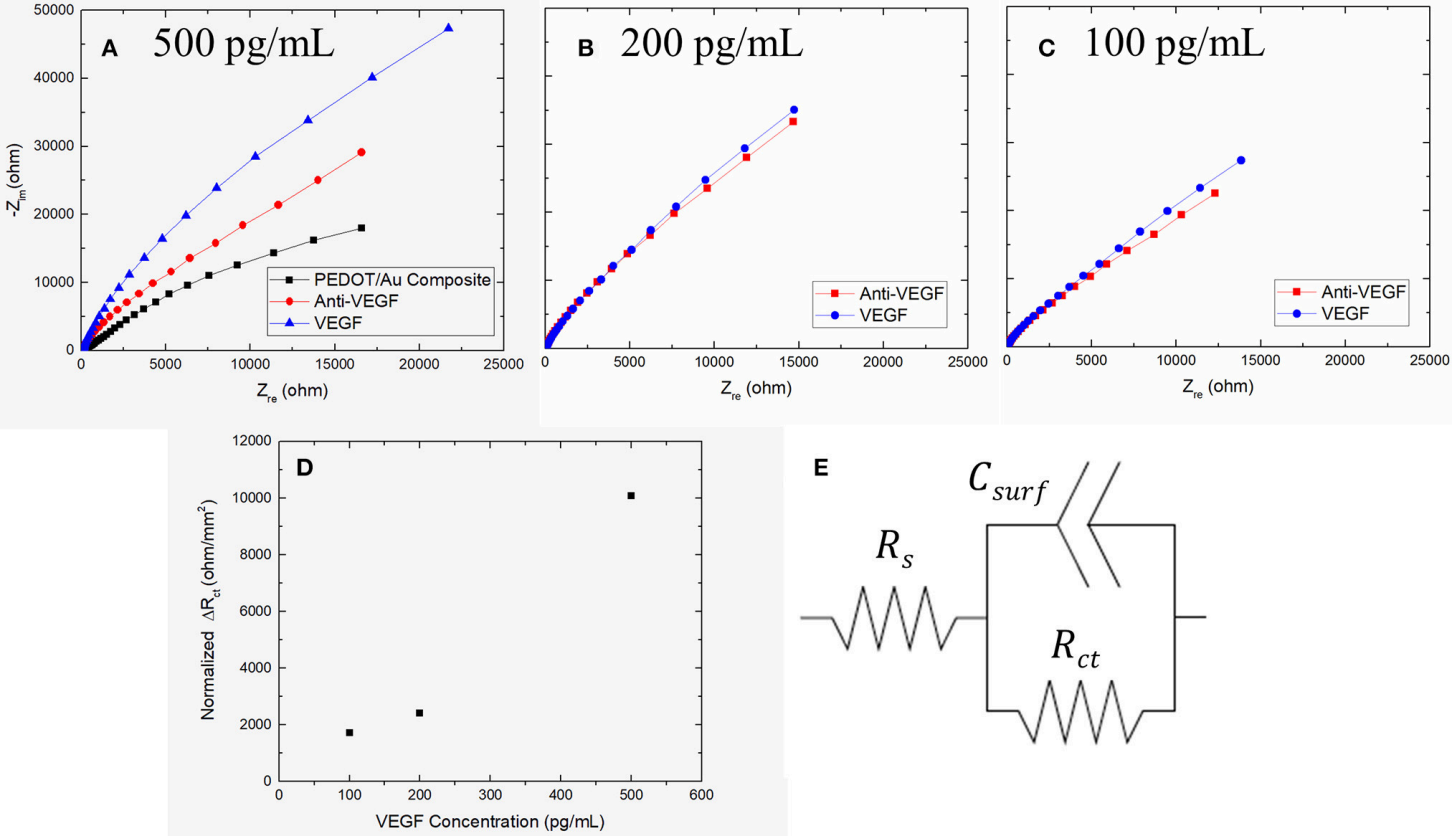
Nyquist plots of impedance spectra obtained from the increasing concentration of VEGF of **(A)** 500 pg/mL **(B)** 200 pg/mL **(C)** 100 pg/mL on three stainless steel electrodes and **(D)** Plot of Δ*R*_*ct*_ as a function of VEGF concentration **(E)** Schematic diagram of an equivalent circuit model.

Figure 2 shows the surface morphology and cross-sectional analysis of a PEDOT/Au NP composite, characterized using SEM/FIB and optical microscopy. An increase in surface roughness with larger grains was observed due to aggregation of Au NPs. A yellowish, rather than reddish color in Figure 2 (a-1) indicated that Au NPs were mostly aggregated inside the film. The thickness of the PEDOT/Au NP composite layers was directly measured from the FIB-SEM images, resulting in an estimated thickness of 700 nm. Interestingly, the size of the aggregated Au NPs was much <100 nm which was barely observable by SEM analysis. The dispersion of Au NPs in the PEDOT matrix was characterized using EDS, TEM, and STEM. In Figure 2c, colored EDS element maps indicate that the aggregated Au NPs were distributed evenly throughout the PEDOT layer and also confirms the presence of primary elements C, O, S, and Au, corresponding to PEDOT and Au NPs, as expected. High magnification images by TEM and STEM (Figure 3D) indicated that the diameter of Au NPs was about 10 nm, again consistent with analysis of the Au NP solution (Figure S1).

### VEGF Sensing Performance of PEDOT/Au NP Composites on a Free-Standing Pad Electrode

AC impedance measurements were carried out over a range of frequencies from 1 Hz to 10 kHz, in the presence of a redox-active electrolyte following each modification step. The first measurements were performed on the free-standing pad electrodes coated with a PEDOT/Au NP composite. These same electrodes were also measured after VEGF incubation followed by anti-VEGF immobilization. Figure 3A shows Nyquist impedance plots after each step. The obtained impedance spectra were fit to an equivalent circuit model using an electrochemical modeling and analysis software ZView, as shown in Figure 3E, where the solution resistance, *R*_*s*_ represents the electrical resistance of the solution bulk between the electrodes, the constant phase element (CPE) is related to a double-layer capacitance, and the charge transfer resistance, *R*_*ct*_is related to the charge transfer rate of the redox reaction at the biofunctionalized electrode (Braiek et al., 2012). We found that a CPE was a better fit to the experimental results, was used instead of a pure capacitor presumably due to anomalous diffusion from the porous surface of a PEDOT/Au NP composite (Sharifi-Viand et al., 2012). The impedance value of *R*_*ct*_ was obtained from each step. The resistive, capacitive, and inductive processes occur in the electrochemical cell during AC impedance measurements (Daniels and Pourmand, 2007). The negative imaginary component of impedance (Z_im_) can be plotted against the real impedance component (Z_re_) at each excitation frequency in what is known as a Nyquist, Cole-Cole, or complex impedance plane plot (Spichiger-Keller, 1998). Nyquist plots of all impedance data consist of depressed semi-circles at the high and middle frequency region and straight lines in the low frequency region. A Warburg impedance exists when the electrochemical reaction rate is limited by the mass transport of redox probes to the electrode surface (Suni, 2008). This is generally observed at relatively low frequencies by an interfacial line at 45 degrees to the real axis. The distinct semicircle at high frequencies and a Warburg impedance at sufficiently low frequencies (e.g., 0.1 Hz) in Nyquist plot indicates that the impedance is controlled by the interfacial electron transfer and by diffusion of the redox probe. The incomplete semi-circle at given frequency range in in Figure 3A indicates a highly resistive surface and high-frequency domain characteristic of an interfacial charge-transfer mechanism due to a typical impedance response for a free-standing pad electrode. The best matching of experimental impedance spectra with Randall's model was obtained in the frequency range from 1 Hz to 10 kHz. The semicircle at the high frequency region has been reported to correspond to the charge-transfer process at the electrode/electrolyte interface (Dutta et al., 2005). Since the diameter of the semi-circle is proportional to *R*_*ct*_, the EIS semi-circle in Figure 3A represents qualitative characteristic of insufficient charge transfer on the surface of stainless steel electrode. The larger diameter of the semi-circle in the presence of biological materials indicates that anti-VEGF and VEGF complexes create an electrostatic barrier to the negatively charged redox couple in solution and effectively hinder the transfer of electrical charges toward the electrode surface (Kharitonov et al., 2000). After anti-VEGF molecules were bound to the electrode modified with a PEDOT-Au NP composite, *R*_*ct*_was significantly increased compared to the previously modified electrode surfaces, apparently due to a surface coverage with an additional insulating layer to hinder the redox reaction of [Fe(CN)_6_]^3−/4−^. The increase in *R*_*ct*_ indicates a successful immobilization of anti-VEGF on the PEDOT/Au NP composite electrode modified with a carboxylic group of Au NPs. Following the incubation of 500 pg/mL of VEGF on the surface of biosensor, *R*_*ct*_was further increased, which confirms that the surface was covered with an extra layer derived from binding of VEGF to anti-VEGF. Figures 3B,C shows Nyquist plots of 100 and 200 pg/mL as a function of VEGF concentration.

Three different samples were tested with increasing VEGF concentrations. The average *R*_*ct*_ from three electrodes after PEDOT/Au NP composite were deposited was 104,000 Ω with a relative standard deviation of 12% shown in Table 1. To construct a calibration curve for the biosensor, the *R*_*ct*_variation was calculated against the value of *R*_*ct*_ of antibody immobilization using equation 1.

(1) $$\begin{array}{r} \Delta R_{ct}=R_{ct}\left(\frac{antiVEGF}{VEGF}\right)-R_{ct}\left(antiVEGF\right) \end{array}$$

**Table 1:** The fitted result of the impedance spectra in Figure 3 obtained from ZView software.

	***R*_*s*_(Ω)**	***R*_*ct*_(Ω)**	***CPE–T***	***CPE–P***
PEDOT/Au	79.0	37,900	6.68E-06	0.848
Anti-VEGF	78.9	88,900	6.08E-06	0.878
500 pg/mL VEGF	73.0	208,600	4.32E-06	0.876
Anti-VEGF	59.2	102,110	4.24E-06	0.856
200 pg/mL VEGF	60.9	130,800	3.56E-06	0.897
Anti-VEGF	63.0	121,450	4.63E-06	0.881
100 pg/mL VEGF	70.0	141,920	4.55E-06	0.875

where $R_{ct}\left(\frac{antiVEGF}{VEGF}\right)$ is the value of *R*_*ct*_when VEGF was bound to anti-VEGF and *R*_*ct*_(*antiVEGF*) is the value of *R*_*ct*_ when anti-VEGF antibody was immobilized on the surface of the electrode. All the values of Δ*R*_*ct*_ summarized in Table 2 were normalized by dividing by the value (11.88 mm^2^) of electrode surface area. It was noted that the slope of Nyquist plots increased with increasing VEGF concentration. Figure 3D shows a linear relationship between *R*_*ct*_and VEGF concentration at 100–500 pg/mL, which is applicable to the average VEGF concentrations required for AMD treatment.

**Table 2:** The normalized Δ*R*_*ct*_ as a function of VEGF concentration.

	**Δ*R*_*ct*_**
100 pg/mL VEGF–Anti VEGF	1723
200 pg/mL VEGF–Anti VEGF	2415
500 pg/mL VEGF–Anti VEGF	10078

As discussed above, the aggregated layer of Au NPs improved sensitivity to impedance. The thickness of PEDOT/Au NP layer was significantly increased with the addition of excess Au solution, which tended to cause delamination of PEDOT/Au film shown in Figure S2 and to decrease the reproducibility of measurements. Previously, Zhou et al. (2010) reported that the thickness of the PEDOT coating was approximately proportional to deposition time and had a direct effect on the mechanical failure observed. Similarly, we observed that more mechanical failures such as cracking and delamination occurred with thicker films, presumably due to a higher stress imposed on the film. Under electrical stimulation conditions, a thick film was observed to form cracks, and delamination, which would limit the use of the same sample for a multiple EIS test. We found that the exposure time to the solution for immobilization of anti-VEGF and VEGF binding had a significant impact on the delamination of PEDOT. When the exposure time was longer than 4 h, there was a significant decrease in the reproducibility of the EIS measurements.

### PEDOT/Au NP Composites on Dot Electrodes

Next, screen-printed dot electrodes (SPEs) were tested in order to improve the sensitivity and homogeneity of the polymer/metal coating. SPEs are low-cost, disposable devices (Hayat and Marty, 2014). The Au SPE design we used consisted of three electrodes, including an Au working, an Au counter, and an Ag reference electrodes. This configuration is ideal for examining small sample volumes as low as 50 μL. The PEDOT/Au NP composites were electrochemically polymerized on the Au SPE electrode under galvanostatic conditions in a mixture of EDOT and Au NPs at a current density of 2 mA/cm^2^. Figure S3 shows inhomogeneous structures of PEDOT/Au NP composite on the working electrode. The thickness varied significantly from the center to the peripheral region. A polymer layer with a thickness of 600 nm was deposited in a peripheral region whereas the thickness of polymer/metal composite coating in the central region was only several nanometers. Similar inhomogeneities of surface roughness were also observed at different current densities, which is presumably related to a non-uniform surface charge distribution. Therefore, we have concluded that SPEs cannot be reliably used for our biosensor.

### VEGF Sensing Performance of PEDOT/Au NP Composite on Interdigitated Micro-Strip Electrodes

**Figure 4:**
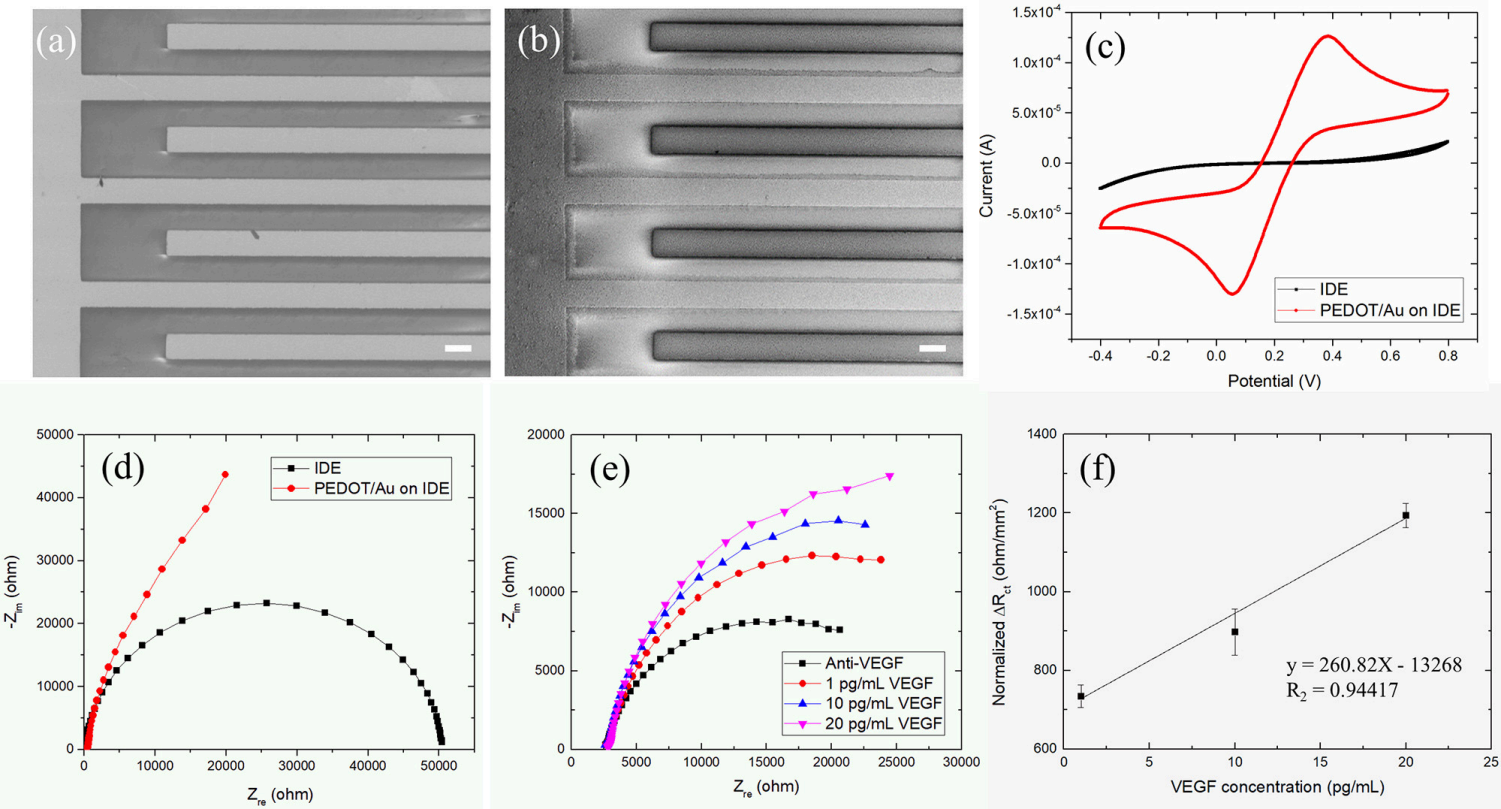
**(a,b)** SEM images (scale bar 10 μm) of a PEDOT/Au NP composite deposited on an IDE, **(c)** Nyquist plots, **(d)** cyclic voltammograms (or CVs) for a bare Au SPE and a PEDOT/Au NP composite-coated IDE **(e)** Nyquist Plot of impedance spectra obtained from the increasing concentration of VEGF from 1 to 20 pg/mL on a Au IDE **(f)** Linear plot of Δ*R*_*ct*_ as a function of VEGF concentration.

Finally, experiments with Au IDEs consisting of two interdigitated micro-strip electrodes with two connection tracks were performed. The interdigitated configuration enhances sensitivity and detection limits of sensors due to the maximized contact area between the sensing layer and a chemical analyte. IDEs have been developed for various sensor applications such as bio sensors (Singh et al., 2011), chemical sensors (Li et al., 2006), and acoustic sensors (Wang et al., 2005). The IDE design including geometrical parameters has been optimized for improved sensitivity of sensors. Min and Baeumner (2004) found that the gap size and finger width of IDE had a significant effect on the signals obtained. Radke et al. (2004) reported that through simulations, the optimal IDE electrode finger width and spacing were 3 and 4 μm, respectively, for enhanced detection of bacteria. Here, commercial IDEs with 10 μm dimensions for bandgaps were tested. The PEDOT/Au NP composites were electrochemically polymerized on Au SPE electrodes under galvanostatic conditions in a mixture solution of EDOT and Au NPs at a current density of 0.1–0.2 mA/cm^2^. The thickness of the thin film was controlled by adjusting the deposition time and the applied electrical charges. The resulting thin films were highly homogenous with an overall thickness of 100–200 nm, as shown in Figures 4a,b. This film showed smooth surfaces with a reduced grain size on the sub-micron scale.

We have tested different current density for three different commercial electrodes since they have different layouts and dimensions. We have limited the maximum deposition time to 1 h for the practical use. Thick PEDOT/Au NP layers seems to be delaminating under prolonged steps but the thin or porous gold layer on the top of sensor deposited at a low current density showed a poor sensitivity to detect VEGF. The applied current density in this paper was chosen to provide denser gold layer and improved sensitivity. It is noted that sensitivity to detect VEGT would likely be improved by further optimization of current density as well as robust surface of electrode. We are planning to investigate further with a needle type electrode.

The electrochemical characteristics before and after the deposition of a PEDOT/Au NP composite on an IDE were characterized by EIS and CV. The change in the diameter of semi-circles in Nyquist plots in Figure 4d can be explained as follows. The qualitatively similar capacitive behaviors were normally obtained from conductive porous structures with 45° inclined line at higher frequencies in the impedance spectra of IDE coated by PEDOT/Au NP composite (Park et al., 2013). The single semi-circle representing the process is controlled by the interfacial electron transfer which was obtained in the impedance spectra of a bare IDE. Figure 4c shows CV obtained with a bare Au IDE (black line) and Au IDE coated with a PEDOT/Au NP composite (red line). The bare Au IDE exhibits a sigmoidal CV curve for the oxidation and reduction of [Fe(CN) _6_]^−3/−4^ whereas a peak-shaped CV with a PEDOT/Au NP composite. This behavior attributes to enhanced mass transport by redox species toward the electrode surface given by geometry characteristic of IDE allowing the current to go to a steady-state value (Yang and Zhang, 2007). The redox potential was measured at +0.06 and +0.38 V for a PEDOT/Au NP composite-coated Au IDE. Compared to a bare IDE, a PEDOT/Au NP composite-coated IDE led to a significant increase in the area within the curve, indicating a larger charge capacity characteristic. Both EIS and CV data demonstrated that a PEDOT/Au NP composite was successfully deposited on the electrode, which is consistent with SEM images in Figure 4b.

Figure 4e shows the complex impedance spectra of the PEDOT/Au NP composite-modified Au IDE as a function of VEGF concentration. The uniform thin single layer of a PEDOT/Au NP composite on an Au IDE allowed for a multiple test on the same sample unlike non-uniform layers on the stainless steel electrode, as discussed earlier. EIS data were fitted to the equivalent circuit model in Figure 4e in the frequency from 0.5 Hz to 1 kHz shown in Table 3. All the values of Δ*R*_*ct*_ were normalized by dividing by the value (12.75 mm^2^) of electrode surface area summarized in Table 4. Figure 4f shows a calibration line of the impedance biosensor encompassing the desired range of detection. It was found that *R*_*ct*_ variation and the logarithmic value of VEGF concentrations have a linear correlation within a range of 1–20 pg/mL which is applicable for VEGF concentrations of average AMD patients. The linear correlation led to a slope of 24.1 and a correlation coefficient of 0.99, and the limit of detection (LOD) was 0.5 pg/mL. To validate the reproducibility of the biosensor, three different electrode samples were measured under the same conditions. The relative standard deviation for three electrodes was 6.5% indicating reasonably good reproducibility of the biosensor. The correlation coefficient in this study is 0.99 ± 0.064. The increase in *R*_*ct*_ with increasing VEGF concentration was discussed earlier. Increased thickness of the bio layers due to binding of VEGF onto the immobilized anti-VEGF resulted in an increase in *R*_*ct*_ to hinder the electron transfer process of redox couples.

**Table 3:** The fitted result of the impedance spectra in Figure 4e obtained from ZView software.

	***R*_*s*_(Ω)**	***R*_*ct*_(Ω)**	***CPE–T***	***CPE–P***
Anti-VEGF	2,579	23,805	6.31E-06	0.776
1 pg/mL VEGF	2,704	33,151	6.89E-06	0.820
10 pg/mL VEGF	2,727	34,379	7.71E-06	0.882
20 pg/mL VEGF	2,889	38,771	7.16E-06	0.900

**Table 4:** The normalized Δ*R*_*ct*_ a function of VEGF concentration.

	**1st**	**2nd**	**3rd**
Δ*R*_*ct*_ (1 pg/mL VEGF–Anti VEGF)	733.02	763.14	705.88
Δ*R*_*ct*_ (10 pg/mL VEGF–Anti VEGF)	829.34	930.03	931.76
Δ*R*_*ct*_ (20 pg/mL VEGF–Anti VEGF)	1173.80	1228.31	1177.09

In this paper, we have used commercial Au NPs with a diameter of 10 nm. Piletsk et al. investigated the correlation between the size of biotinylated nanoparticles and their affinity in relation to interactions with the solid surface (Piletska and Piletsky, 2010). It was shown that the increase in the particle size from 50 to 200 nm reduced the affinity of biotin–streptavidin interactions so that sensitivity decreased from 1.2 × 10^−12^ to 1.2 × 10^−10^ M (Piletska and Piletsky, 2010). Similarly, we expect that biofunctional Au NPs of smaller sizes increase the active sites for anti-VEGF immobilization at the gold surface and the higher concentrations of immobilized anti-VEGF result in stronger binding with VEGF.

We have focused on detecting VEGF-165 as a biomarker of AMD, which could also be applied to detect other VEGF isoforms. To immobilize VEGF-165, we have used ranibizumab which was produced using recombinant antibody technology. Ranibizumab also bounds all isoforms of VEGF-A but markedly low affinity (Papadopoulos et al., 2012). Aflibercept is another VEGF antibody which has a higher all isoforms of VEGF-A binding affinity as well as its ability to bind VEGF-B and PlGF (Mantione et al., 2017). However, Aflibercept is larger molecules than Ranibizumab, which might result steric hindrances during immobilizing on the sensor surface.

## Conclusions

We have designed and characterized a label-free impedance biosensor using PEDOT/Au NP composites for detecting VEGF-165. As a proof of principle, PEDOT/Au NP composites were galvanostatically electropolymerized onto the three types of electrodes including stainless steel free-standing pads, Au SPEs, and Au IDEs. Thin films of a PEDOT Au NP composites with uniform thickness were successfully deposited on Au IDEs whereas inhomogeneous layers with the potential to delaminate were found on both on stainless steel electrodes and Au SPEs, resulting in a low reproducibility of the resulting biosensors. Nyquist plots obtained from the use of IDEs showed a significant change in *R*_*ct*_ due to anti-VEGF immobilization and its specific binding to VEGF. For a biosensor with IDEs, *R*_*ct*_ was found to have a linear relationship with VEGF over a concentration range of 1–20 pg/mL. The performance of the biosensor was further investigated in terms of limit of detection and reproducibility. A detection limit of 0.5 pg/mL and correlation coefficient of 0.987 were obtained with a relative standard deviation of 0.99 ± 0.064 were obtained. Our work demonstrates the potential of PEDOT/Au NP composites as effective materials for reproducible and sensitive measurements of VEGF concentration. Future experiments will seek to further extend this biosensor technology to *in-vivo* testing, perhaps using needle type electrodes.

## Author Contributions

DM and Rl conceived of the idea to use conjugated polymers as the basis for VEGF sensors. The development of specific methods, in particular including the use of metal NPs and the experimental work, were done by MK. The paper was written by MK, with editorial input and final editing by all co-authors. The final draft was prepared and uploaded by DM.

### Conflict of Interest Statement

The authors declare that the research was conducted in the absence of any commercial or financial relationships that could be construed as a potential conflict of interest.
