# Supplementary material for: Impedimetric Biosensors for Detecting Vascular Endothelial Growth Factor (VEGF) Based on Poly(3,4-ethylene dioxythiophene) (PEDOT)/Gold Nanoparticle (Au NP) Composites
Source: Front Chem. 2019 Apr 16;7:234. doi: 10.3389/fchem.2019.00234 (PMC6477177; doi:10.3389/fchem.2019.00234)
Supplement: Supplementary file 1 [file Table_1.DOCX]

**Supplementary Materials**

**
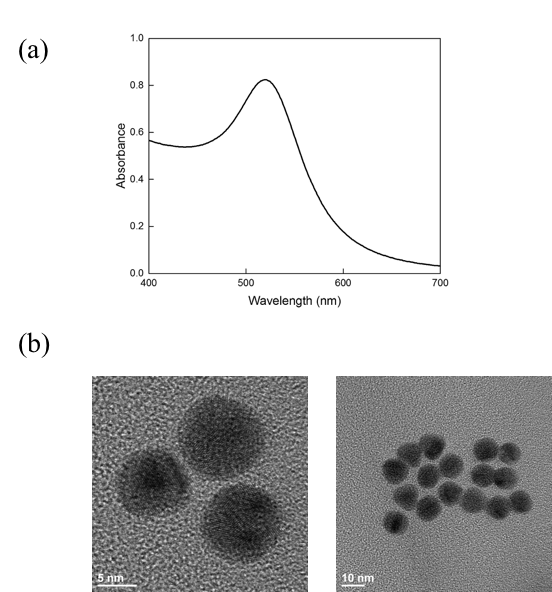
**

**Figure S1**. (a) UV-Vis spectrum and (b) TEM images of Au NPs

**

**

**Figure S2**. SEM image of delaminated top surface of a PEDOT-Au NP film on a stainless steel electrode





600 nm

**Figure S3**. SEM images of a SPE (left) and a schematic diagram of non-uniform polymer film structure (right)
